# Elastic Properties of Taurine Single Crystals Studied by Brillouin Spectroscopy

**DOI:** 10.3390/ijms22137116

**Published:** 2021-07-01

**Authors:** Dong Hoon Kang, Soo Han Oh, Jae-Hyeon Ko, Kwang-Sei Lee, Seiji Kojima

**Affiliations:** 1 Nano Convergence Technology Center, School of Nano Convergence Technology, Hallym University, 1 Hallymdaehakgil, Chuncheon 24252, Korea; rkdehdgns99@naver.com (D.H.K.); soohanoh@naver.com (S.H.O.); 2 Center for Nano Manufacturing, Department of Nano Science & Engineering, Inje University, Gimhae 50834, Korea; kwangsei28@hanmail.net; 3 Division of Materials Science, University of Tsukuba, Tsukuba 305-8573, Japan; kojima@ims.tsukuba.ac.jp

**Keywords:** taurine, elastic constant, Brillouin scattering, acoustic mode

## Abstract

The inelastic interaction between the incident photons and acoustic phonons in the taurine single crystal was investigated by using Brillouin spectroscopy. Three acoustic phonons propagating along the crystallographic *b*-axis were investigated over a temperature range of −185 to 175 °C. The temperature dependences of the sound velocity, the acoustic absorption coefficient, and the elastic constants were determined for the first time. The elastic behaviors could be explained based on normal lattice anharmonicity. No evidence for the structural phase transition was observed, consistent with previous structural studies. The birefringence in the *ac*-plane indirectly estimated from the split longitudinal acoustic modes was consistent with one theoretical calculation by using the extrapolation of the measured dielectric functions in the infrared range.

## 1. Introduction

Taurine (2-aminoethanesulfonic acid), NH_3_(CH_2_)_2_SO_3_, is a well-known and important organic compound that is found in many organs of animal bodies, the bile being the most representative one [1]. Taurine is one of the sulfur-containing amino acids, but it does not belong to the constituents of proteins and is thus regarded as a nonessential amino acid. However, taurine is known to have many biological functions, such as membrane stabilization [2], osmoregulation [3], and antioxidation [4], to name a few. Moreover, taurine has been studied for various applications, such as sensors [5], nanofiltration [6], etc.

On the other hand, fundamental aspects of taurine have been investigated by various methods because taurine is a model system of a sulfur-included organic compound. In particular, a single crystal form or a powder pellet has been used for the characterization of the exact structure and vibrational properties. Taurine can be formed into a colorless crystal and can thus be studied as one of the organic crystals. The structure of the taurine crystal was first determined by X-ray scattering to be monoclinic *P2_1_/c* [7,8]. There are trans and gauche forms in taurine molecules, but the taurine crystal is grown in the zwitterion configuration (NH_3_^+^–CH_2_–CH_2_–SO_3_^−^) where only the gauche configuration exists, as confirmed by x-ray crystallography [8,9,10,11,12]. According to these structural studies, the N—H···O hydrogen bonds form a three-dimensional network, and the electrostatic potential exhibits large negative and positive regions near SO_3_^−^ and NH_3_^+^, respectively.

There are several vibrational studies on taurine material in its various forms, including solutions to solid states [13,14,15,16,17,18,19,20,21,22]. These studies, combined with normal mode analysis, revealed detailed vibrational modes of taurine molecules, power samples, and single crystals. Some of these vibrational studies include a surface-enhanced Raman study carried out on taurine absorbed on silver nanoparticles [19] and the combination of infrared and Raman analyses for taurine single crystals [22]. In particular, temperature- or pressure-dependent spectroscopic investigations are a promising tool for studying any possible phase transitions in these organic materials.

Taurine crystal shows successive pressure-induced phase transitions at ~0.7 and ~5.2 GPa, as revealed by high-pressure Raman spectroscopy [15]. A high-temperature Raman study above room temperature did not reveal any high-temperature phase transition [18]. On the other hand, Lim et al. suggested a first-order phase transition at ~250 K (measured upon cooling) by using a temperature-dependent Raman study below room temperature [16]. They also presented an enthalpic anomaly in a similar temperature range where the phase transition temperature showed a large thermal hysteresis of about 21 K. On the other hand, Bajaj et al. reported a low-temperature infrared study of torsional vibrations of taurine [21]. They observed a splitting of the CSH torsional mode near 250 K consistent with the Raman study described above.

However, apart for these two spectroscopic studies, there has been no other report about the structural phase transition of taurine single crystals. Moreover, the crystal structure (space group) does not change below room temperature down to 120 K [23]. Other structural studies also confirmed that the space group at low temperatures below 250 K is *P2_1_/c*, the same as that measured at room temperature [10,11]. In this context, the existence of a low-temperature phase transition near 250 K and its exact structure below this temperature are still controversial.

The purpose of the present study is aimed at tackling this issue by using Brillouin spectroscopy, which is one of the inelastic light-scattering methods. Brillouin light-scattering is based on the inelastic interaction between the incident photons and acoustic phonons in the crystal. From the measured inelastic photon signals, sound velocities and elastic stiffness coefficients (abbreviated as elastic constants) can be derived [24]. Elastic constants are directly associated with interatomic and intermolecular potentials and are thus affected by phase transitions [25]. Brillouin spectroscopy has been applied to various organic crystals including pharmaceutical materials, such as aspirin [26,27], ibuprofen [28], acetaminophen [29], ketoprofen [30], indomethacin [31], etc. Some of these studies revealed detailed temperature dependences of elastic constants and acoustic damping effects in these organic crystals. The present study is the first report on the temperature dependence of the elastic properties of taurine single crystals. We could not find evidence for a structural phase transition in the investigated temperature of −185 °C ~ 175 °C.

## 2. Materials and Methods

The powder of taurine (purity ≥ 99%) was purchased from Aldrich and was dissolved in doubly distilled water. Single crystals of taurine were grown from an aqueous solution by slow evaporation technique at about 38 °C, the form of the resulting crystals depending on the rate of evaporation or supersaturation of the solution, as mentioned in previous works [7,32,33,34]. Optically transparent crystals have a different morphology ranging from a prism shape to acicular shape, in agreement with references [7,32,33,34]. With a rapid evaporation or a high degree of supersaturated solution, needle-shaped crystals were formed, the length of the needle being parallel to the *a*-axis. On the other hand, with a slow evaporation or a low degree of supersaturated solution, the crystals adopted a tabular form, the greatest length being along the *a*-axis. The monoclinic *b*-axis is perpendicular to the largest face and the *a*-direction [32,33]. Thus, the natural plane perpendicular to the *b*-axis was chosen for the investigation.

**Figure 1:**
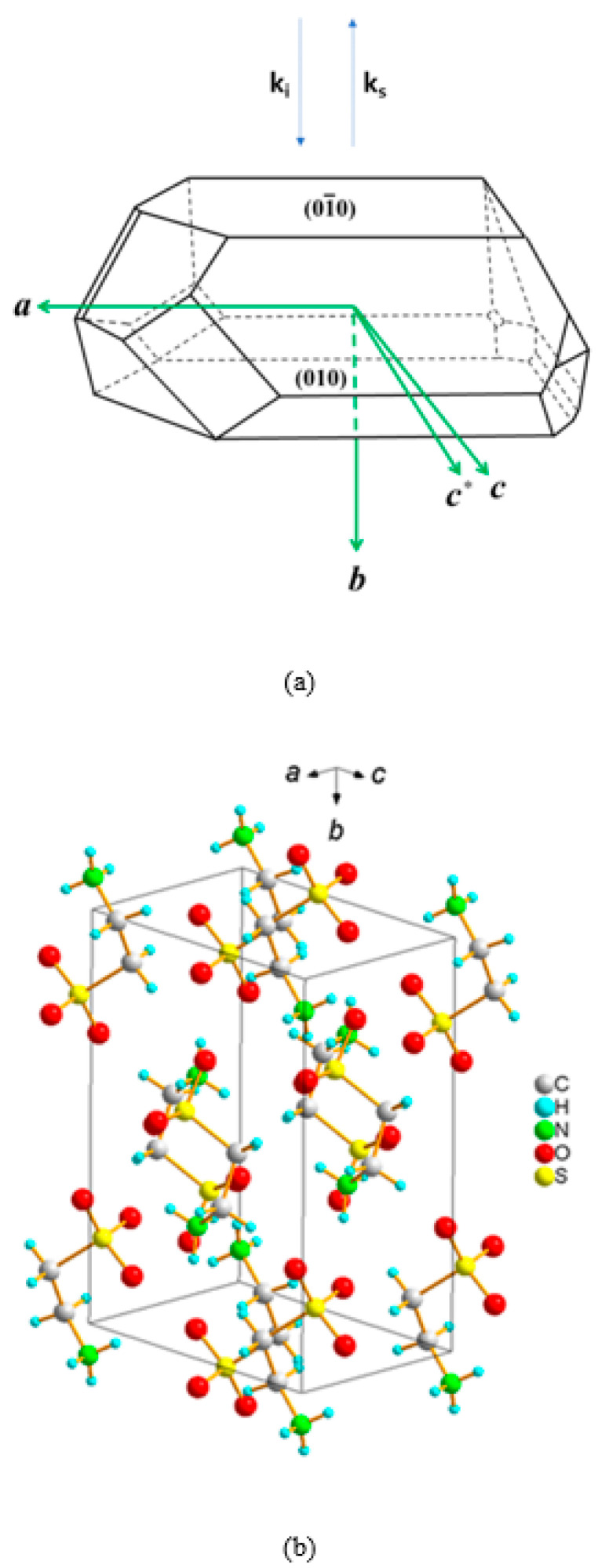
(**a**) The crystal morphology along with major crystallographic surfaces and axes. Two vectors **k_i_** and **k_s_** denote the wave vectors of the incident and the scattered lights, respectively. c* direction is perpendicular to both *a* and *b* axes. (**b**) The crystallographic unit cell of the taurine single crystal.

Figure 1a,b shows the morphology of the taurine single crystal along with the crystallographic axes and the crystallographic unit cell, respectively. The directions of the incident and the scattered light were denoted in terms of the wave vectors **k_i_** and **k_s_**, respectively. A conventional Brillouin scattering spectrometer (TFPI-1, JRS Co., Zürich, Switzerland) was used to perform the measurements. Sample excitation was achieved by using a diode-pumped solid-state laser with a wavelength of 532 nm. The sample was put in a cryostat stage (THMS 600, Linkam, Tadworth, England) for temperature variation. All measurements were carried out upon cooling. An optical microscope (BX41, Olympus, Tokyo, Japan) with an objective lens of ×20 magnification was employed for backscattering geometry along the monoclinic *b* axis, as shown in Figure 1. The polarization direction of the incident light was in the *ac* plane, and no analyzer was used for the scattered light. Before every measurement, the temperature was stabilized for at least 2 min.

## 3. Results and Discussion

**Figure 2:**
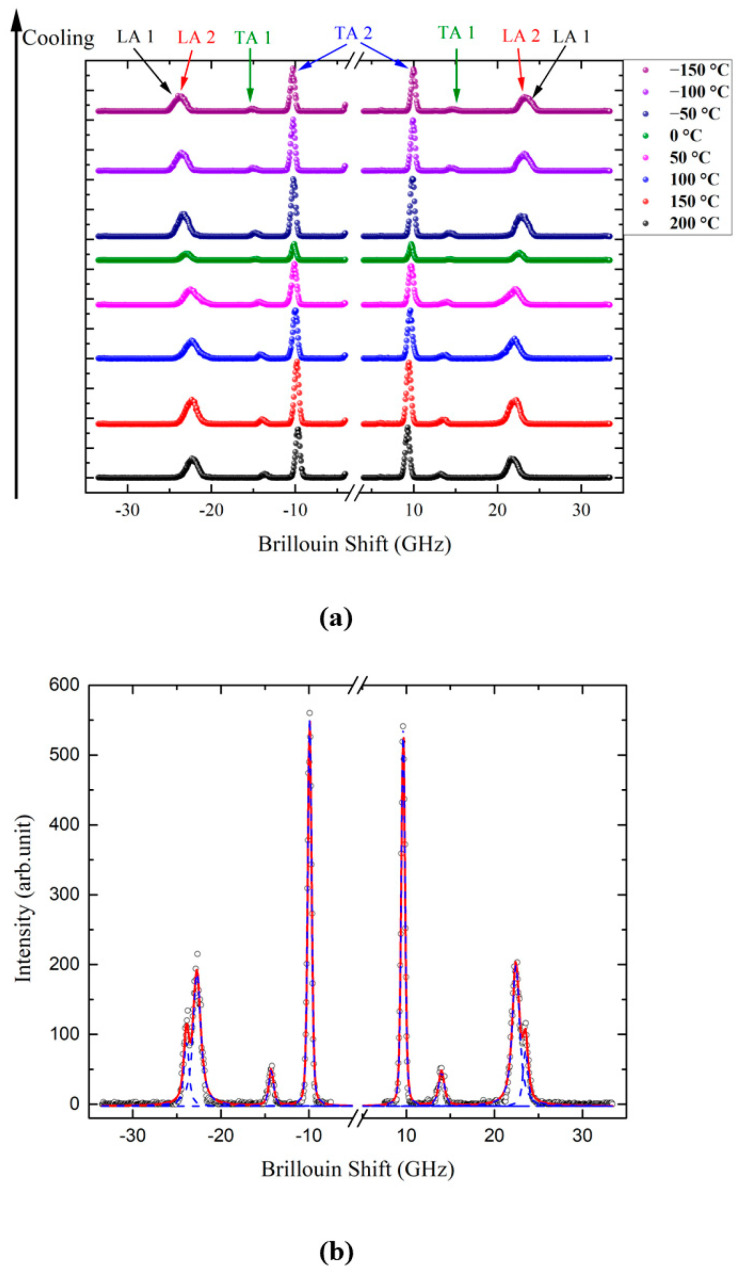
(**a**) Brillouin spectra of a taurine crystal at a few temperatures. (**b**) The Brillouin spectra of a taurine crystal measured at 30 °C and the fitting lines for each acoustic mode.

**Figure 3:**
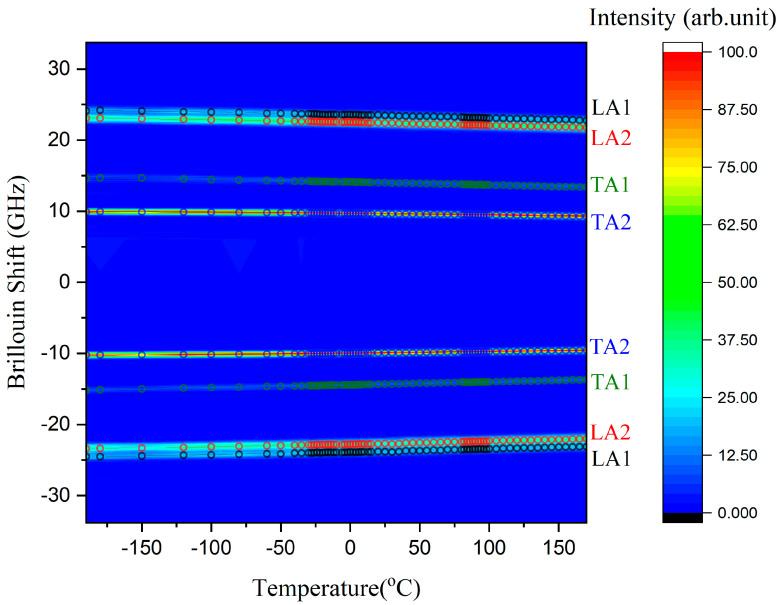
The intensity color plot of the measured Brillouin spectra as a function of the temperature. Mode frequencies are denoted in terms of circle symbols.

Figure 2a shows the Brillouin spectra of the taurine single crystal at several temperatures. Each Brillouin spectrum consists of one longitudinal acoustic (LA) mode at ~24 GHz and two transverse acoustic (TA) modes near 15 and 10 GHz. However, a close inspection shows that the LA mode shows splitting at all temperatures. Figure 2b shows one example of the curve-fitting result for the spectrum measured at 30 °C. Splitting of the LA mode has been observed from several birefringent single crystals, such as aspirin and 1,2,4,5-tetrabromobenzene single crystals [26,35]. Thus, the most probable origin of the splitting of the LA mode is the birefringence of the taurine single crystal, which the incident polarization feels. Another possible origin for the splitting is the possible existence of monoclinic twins, where different twin structures might contribute to the splitting of the LA mode. The half widths do not show noticeable changes, but the mode frequencies seem to exhibit a slight hardening upon cooling. Figure 3 shows the intensity color plot of the measured Brillouin spectra as a function of the temperature. It includes the traces of the mode frequencies of two LA modes (LA1 and LA2, denoted with descending frequencies) and two TA modes (TA1 and TA2, denoted with descending frequencies), all being denoted by circle symbols. All acoustic modes seem to become hardened upon cooling.

Each Brillouin spectrum was curve-fitted by using the Voigt function, which is a convolution of a Lorentzian function (an approximate form of the response function of the damped harmonic oscillator for the phonon modes) and a Gaussian function (an instrumental function of the interferometer which causes a broadening of the spectrum). The Brillouin frequency shift (*ν*_B_) corresponding to the mode frequency and the full width at half maximum (FWHM, *Γ*_B_) of each acoustic mode could be obtained as a function of the temperature. It clearly shows a splitting of the LA mode. There seems to be no central peak within the measured frequency range.

**Figure 4:**
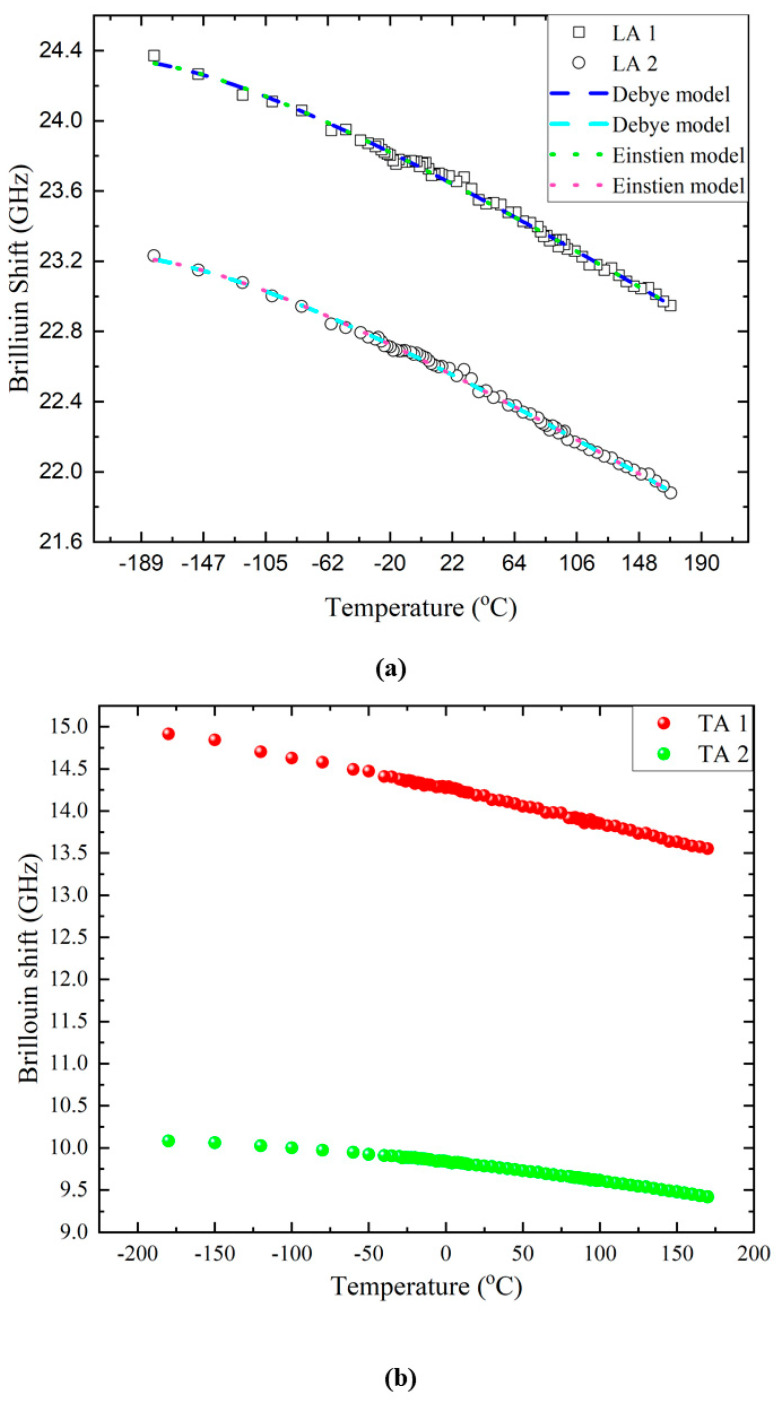
Temperature dependences of the Brillouin frequency shift of (**a**) the LA modes and (**b**) the TA modes. The dotted and dashed lines in (**a**) denote the best-fitted results by the Debye and the Einstein models. See the text for details.

Figure 4 shows the temperature dependences of the Brillouin shifts of the acoustic modes. The split LA modes, as well as the two TA modes, show hardening upon cooling without any noticeable anomalies in the investigated temperature range. When there is a phase transition in condensed matters, one may observe various spectral changes in the light-scattering spectrum, such as the formation of strong central peaks in order-disorder phase transitions [36] or low-frequency soft optic modes [37], which may couple to the acoustic modes, resulting in substantial acoustic anomalies. These anomalies are in general associated with a downward softening or sudden, discontinuous changes in the mode frequencies concomitant with a significant increase in the half widths near or upon approaching the phase transition point [38]. The present result indicates that there is no experimental evidence for elastic anomalies associated with structural phase transitions in taurine single crystals at the present scattering geometry in the investigated temperature range of −185 °C to 175 °C. This result is in contrast to the result of the temperature-dependent Raman study where the authors suggested that a first-order phase transition occurs at −23 °C [16].

The monotonic increase in the mode frequency upon cooling is conventionally observed from normal solids which do not show any structural phase transition. In this case, normal lattice anharmonicity plays a critical role in the change of acoustic properties. There are two theoretical models and corresponding functions to describe the temperature dependence of the acoustic mode frequency: the Varshni function based on the Einstein model [39] and the Lakkad function based on the Debye approach [40]. When the Einstein model is adopted, the temperature dependence of the Brillouin shift is expressed as:

(1) $$\nu_{B}\left(T\right)=\nu_{0}^{E}\sqrt{1-\frac{a}{\exp\left(\frac{\Theta_{E}}{T}\right)-1}}$$

where $\nu_{0}^{E},\;a,\;\mathrm{and}\;\Theta_{E}$ are the fitting parameters. The $\Theta_{E}$ is called the Einstein temperature. Lakkad’s approach is based on the Debye model, and the Brillouin shift is described by the following equation:

(2) $$\nu_{B}\left(T\right)=\nu_{0}^{D}\sqrt{1-b\Theta_{D}F\left(T/\Theta_{D}\right)}$$

where

(3) $$F\left(T/\Theta_{D}\right)=3{\left(\frac{T}{\Theta_{D}}\right)}^{4}\int_{0}^{\Theta_{D}/T}\frac{x^{3}}{e^{x}-1}dx$$

is the Debye function and $\nu_{0}^{D},\;b,\;\mathrm{and}\;\Theta_{D}$ are the fitting parameters. The $\Theta_{D}$ is called the Debye temperature. The $\nu_{0}^{E}$ and $\nu_{0}^{D}$ in each model are the mode frequencies extrapolated to 0 K. The ratio of $\Theta_{E}$ to $\Theta_{D}$ is known to be 0.75 [41].

The temperature dependences of the mode frequencies of the LA1 and LA2 modes were curve-fitted by using either Equation (1) or Equation (2). The dotted and dashed lines in Figure 4a denote the best-fitted results obtained by the Debye and the Einstein models described above. There is no noticeable difference between the two fitting results. Table 1 shows the obtained best-fitted parameters. It shows that the Einstein temperature is approximately 107 °C (~380 K) while the Debye temperature is near 237 °C (~510 K) in both modes. Moreover, the ratio of $\Theta_{E}$ to $\Theta_{D}$ is 0.74 in both cases, close to the theoretical prediction of 0.75 [41]. All these results indicate that the acoustic mode behavior in taurine single crystals is dominated by the usual lattice anharmonicity frequently observed in normal solids and that there is no direct experimental evidence for elastic anomalies associated with any structural phase transition in the investigated temperature range.

The mode frequency and the FWHM measured in the backscattering geometry can be used to calculate the sound velocity (*V*) and the absorption coefficient (*α*) via the following equations:

(4) $$V=\frac{\lambda \nu_{B}}{2n},$$

(5) $$\alpha =\frac{\pi \Gamma_{B}}{V}.$$

In these equations, *λ* and *n* denote the wavelength of the excitation laser light and the refractive index, respectively. For the calculation, the temperature dependence of the refractive index is necessary. However, only the average refractive index of 1.52 (space-averaged one) and the average refractive index of 1.545 on the *ac*-plane were reported experimentally [22]. Thus, the two LA mode frequencies were averaged to get the average mode frequency, which was used to calculate the average sound velocity and the absorption coefficient by using the average refractive index on the *ac*-plane (1.545).

**Figure 5:**
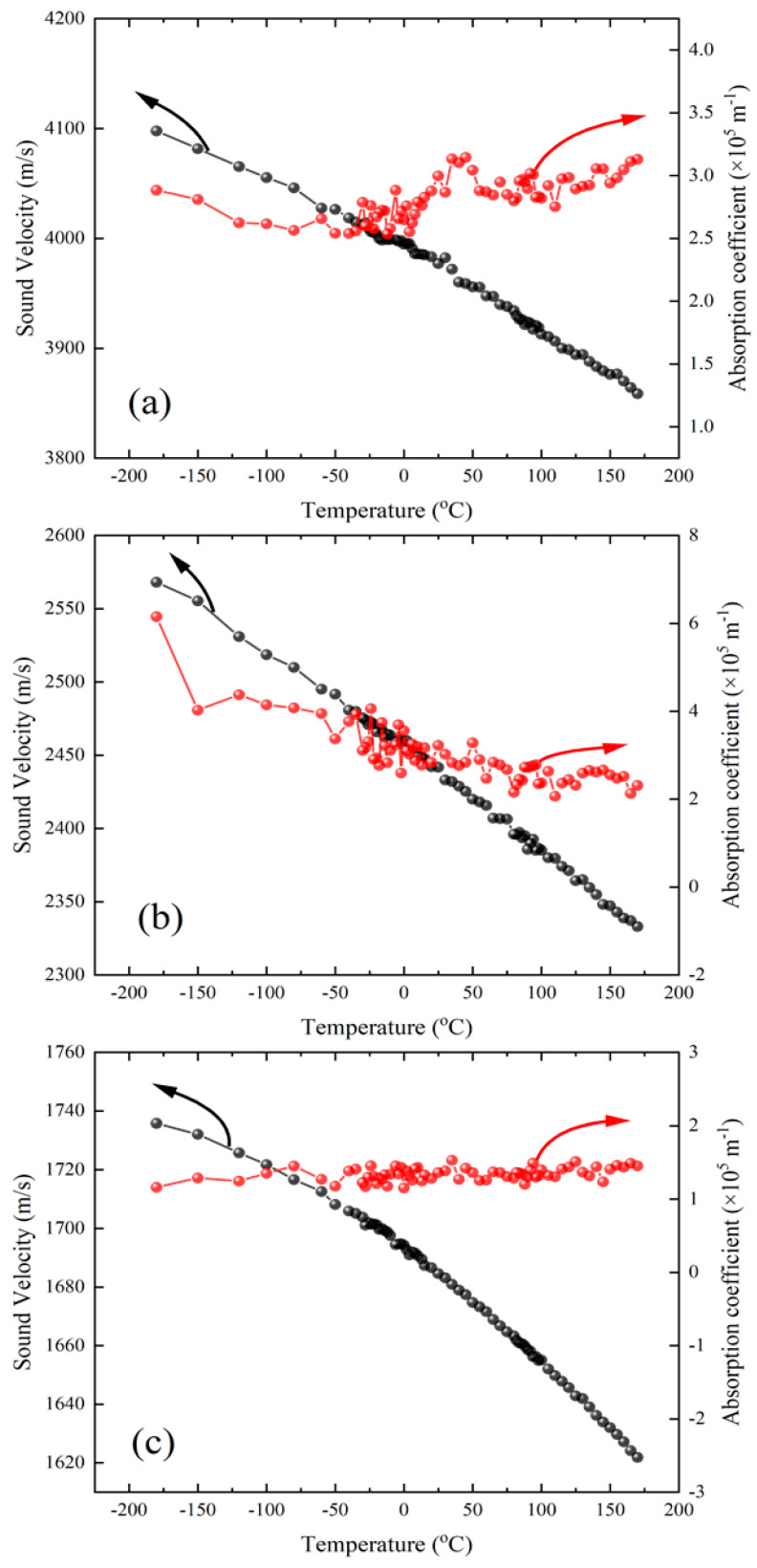
Temperature dependence of the sound velocity and the absorption coefficient of (**a**) the LA mode, (**b**) the TA1 mode, and (**c**) the TA2 mode.

Figure 5a–c shows the calculated sound velocities and the absorption coefficients of the LA and the two TA modes, respectively, as a function of the temperature. The longitudinal sound velocity increases from ~3860 m/s at 170 °C to ~4100 m/s at −180 °C upon cooling. The absorption coefficient of the LA waves is approximately 3 × 10^5^ m^−1^ and does not show any substantial temperature dependence. The transverse sound velocities of the two TA modes show a monotonic increase upon cooling over the whole measured temperature range. The absorption coefficient is nearly constant in both cases. These results demonstrate that the investigated acoustic modes propagating along the [10] direction are not associated with a structural phase transition in the taurine single crystal over the temperature range of −185 °C to 175 °C.

Moreira et al. predicted the theoretical refractive indices *n*_1_ = 1.601, *n*_2_ = 1.597, and *n*_3_ = 1.524 by using the extrapolation of the measured dielectric functions in the infrared range [22]. Since the sound velocity depends on the refractive index via Equation (4), it is worth checking the reliability of the calculated refractive indices of the taurine single crystal from the present study. The polarization of the incident light on the taurine crystal was in the *ac*-plane, and, thus, the two relevant refractive indices are *n*_1_ and *n*_3_. The sound velocities of the LA1 and LA2 modes were calculated by using these predicted refractive indices and Equation (4). The obtained results are shown in Supplementary Figure S1 in the Supplementary Materials. The two sound velocities are nearly the same within 0.21% on average, which indicates that the theoretical prediction for the refractive indices suggested by Moreira et al. [22] is reliable, at least from the viewpoint of the birefringence in the *ac*-plane.

**Figure 6:**
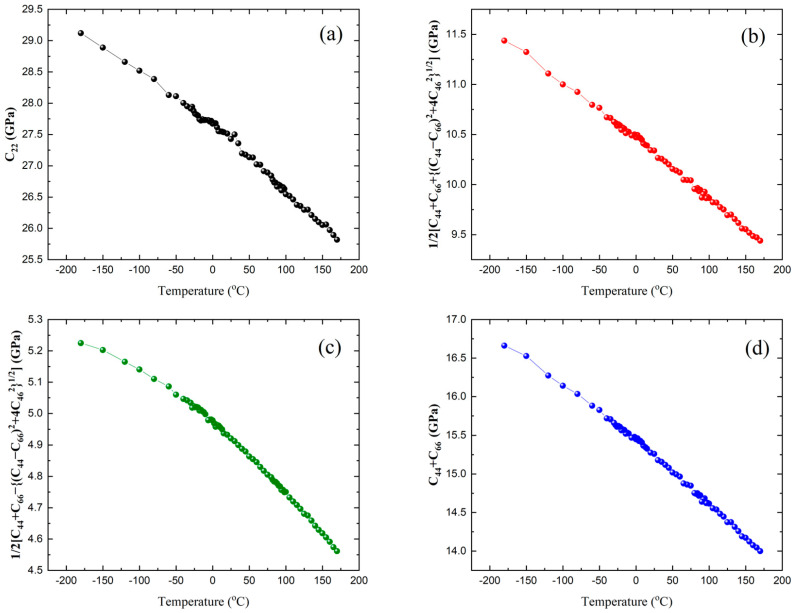
Temperature dependence of (**a**) $C_{22}$, (**b**) $\left(C_{44}+C_{66}+\sqrt{{\left(C_{44}-C_{66}\right)}^{2}+4C_{46}^{2}}\;\right)$/2, (**c**) $\left(C_{44}+C_{66}-\sqrt{{\left(C_{44}-C_{66}\right)}^{2}+4C_{46}^{2}}\;\right)$/2, and (**d**) $C_{44}+C_{66}$.

The elastic constants of the three acoustic modes can be calculated from the obtained sound velocities via *ρV*^2^, where *ρ* is the crystal density. The reported density of 1734 kg/m^3^ [11] was used for the calculation. Table 2 shows the relationship between the acoustic mode and its associated elastic constant in the monoclinic phase [42]. The LA mode corresponds to *C*_22_, while the two TA modes are related to two complex combinations of elastic constants. If the two elastic constants of the two TA modes are added, (*C*_44_ + *C*_66_) can be derived. Figure 6a–c shows the temperature dependences of the three elastic constants shown in Table 2.

**Figure 7:**
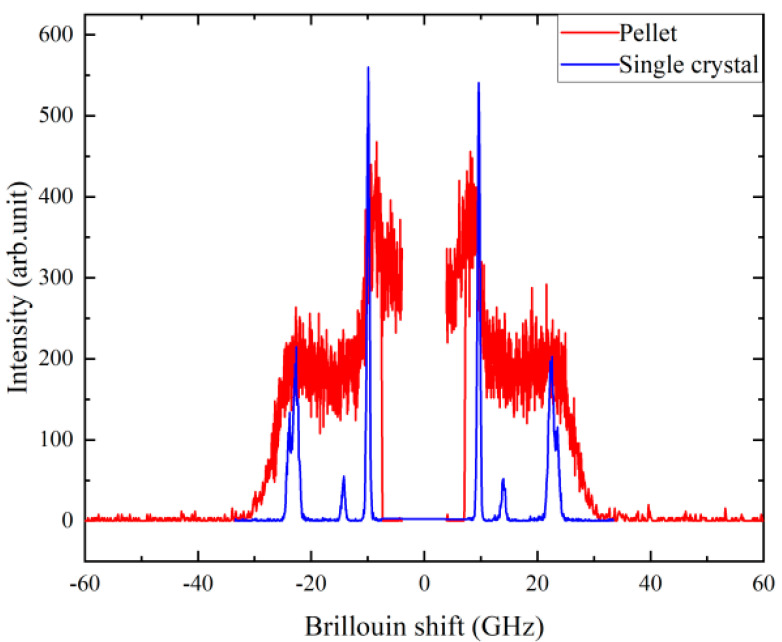
Comparison of the Brillouin spectrum of taurine pellet and single-crystal samples measured at room temperature.

*C*_22_ is 27.5 GPa at room temperature, while the two elastic constants of the two TA modes are 10.3 and 4.9 GPa, resulting in (*C*_44_ + *C*_66_) = 15.2 GPa. There is only one earlier report on the elastic constants of taurine crystals [32]. The *C*_22_ reported in this study is substantially different from this earlier value (~46.9 GPa), while (*C*_44_ + *C*_66_) is similar ((*C*_44_ + *C*_66_) in [32] is ~17.2 GPa). The origin of this large discrepancy for *C*_22_ is not clear at the moment, but it might be due to different experimental techniques having different probe frequencies and/or to different definitions of the crystal orientation. To check the reliability of the large value for *C*_22_ (~46.9 GPa) reported in [32], we made a taurine pellet sample by grinding the crystal into fine powders and pressing them into a circular plate. In the case of pellets and ceramics, the Brillouin light-scattering spectrum consists of continuously varying components whose scattering angle changes between 0 and 180° [43,44]. The Brillouin shift corresponding to the value of 46.9 GPa can be theoretically estimated to be about 31 GHz if we use the average refractive index of 1.574 [22]. This Brillouin shift corresponds to a longitudinal sound velocity of ~5200 m/s, which is unusually large for organic crystals like taurine. Figure 7 shows the comparison of the Brillouin spectrum of the taurine pellet with that of the single crystal measured at room temperature. Interestingly, the main peaks in the pellet spectrum are coincident with those of the single crystals measured at the present backscattering geometry. The broadening of the peaks in the pellet spectrum is due to the overlap of various acoustic modes with changing wave vectors. If the *C*_22_ value of ~46.9 GPa is correct, we should expect some significant contribution (or resonance peak) at ~31 GHz, where only the end of the tail of the main peak at ~23 GHz can be seen. Considering all these aspects, the previous report about the elastic constants of taurine [32] should be revisited.

The strong hydrogen bond network exists in the *ab* plane, with tightly-locked molecules being confined in this plane [23]. On the other hand, the molecules are loosely packed along the *c*-axis. Thus, although we have not investigated *C*_33_ in this study due to the limited available crystal size, we expect that *C*_33_ may be much smaller than *C*_22_. Since elastic properties are important physical parameters required for various applications, such as pharmaceuticals or sensor applications, the present study and the succeeding studies will supply systematic elastic parameters which would accelerate the application of taurine molecules in various fields.

Lima et al. suggested that a structural phase transition existed near 250 K, where some changes in the Raman mode frequency occurred accompanied by enthalpic anomaly [16]. However, structural studies did not reveal any change in the space group of the taurine crystals. The acoustic anomalies and three sets of elastic constants showed monotonic increases upon cooling, which could be explained based on the normal anharmonic effect. Thus, the present study does not support the existence of low-temperature structural phase transitions in taurine single crystals. However, further studies based on other experimental techniques with different spatiotemporal probe scales are necessary in order to unambiguously settle this issue completely.

## 4. Conclusions

In summary, taurine single crystals were grown and investigated by Brillouin light scattering. One LA and two TA modes were observed, and their mode frequencies increased monotonically upon cooling from 175 °C to −180 °C. The temperature dependence of the LA mode could be satisfactorily explained by using both the Debye and the Einstein anharmonic models. The Einstein and Debye temperatures were 380 and 510 K, respectively, with a ratio of 0.74 consistent with theoretical predictions. The temperature dependences of the sound velocities, acoustic absorption coefficients, and associated elastic constants of taurine single crystals could be obtained for the first time. There was no noticeable anomaly in these acoustic properties, which does not support the existence of structural phase transitions suggested by Raman and calorimetric measurements in [16]. The birefringence of the split LA mode could be estimated and was consistent with the theoretical calculations of the three principal refractive indices [22].

## Figures and Tables

**Table 1:** The best-fitted results for the LA modes by using two models as described in the text.

	Einstein Model			Debye Model			$\Theta_{E}/\Theta_{D}$
	$\nu_{0}^{E}$ (GHz)	*a*	$\Theta_{E}$ (K)	$\nu_{0}^{D}$ (GHz)	*b*	$\Theta_{D}$ (K)	
LA 1	24.4	0.151	377	24.4	4.03$\;\times \;10^{-4}$	508	0.74
LA 2	23.2	0.154	382	23.3	4.07 $\;\times \;10^{-4}$	514	0.74

**Table 2:** The correlation between the three acoustic modes and the elastic constants obtained from the present scattering geometry. The numerical values are measured at 20 °C.

Acoustic Modes	Elastic Constant	Elastic Constant (GPa)
L [010]	$C_{22}$	27.5
T1 [100]	$\frac{1}{2}(C_{44}+C_{66}+\sqrt{{\left(C_{44}-C_{66}\right)}^{2}+4C_{46}^{2}}\;$)	10.3
T2 [001]	$\frac{1}{2}(C_{44}+C_{66}-\sqrt{{\left(C_{44}-C_{66}\right)}^{2}+4C_{46}^{2}\;}$)	4.9
T1+T2	$C_{44}+C_{66}$	15.2

## Data Availability

The data presented in this study is contained within the aricle.

## Supplementary Materials

Supplementary materials can be found at https://www.mdpi.com/article/10.3390/ijms22137116/s1.
