# Supplementary material for: Elastic Properties of Taurine Single Crystals Studied by Brillouin Spectroscopy
Source: Int J Mol Sci. 2021 Jul 1;22(13):7116. doi: 10.3390/ijms22137116 (PMC8267836; doi:10.3390/ijms22137116)
Supplement: Supplementary file 1 [file ijms-22-07116-s001.zip › ijms-1265555-supplementary.pdf]

# Supplementary Materials

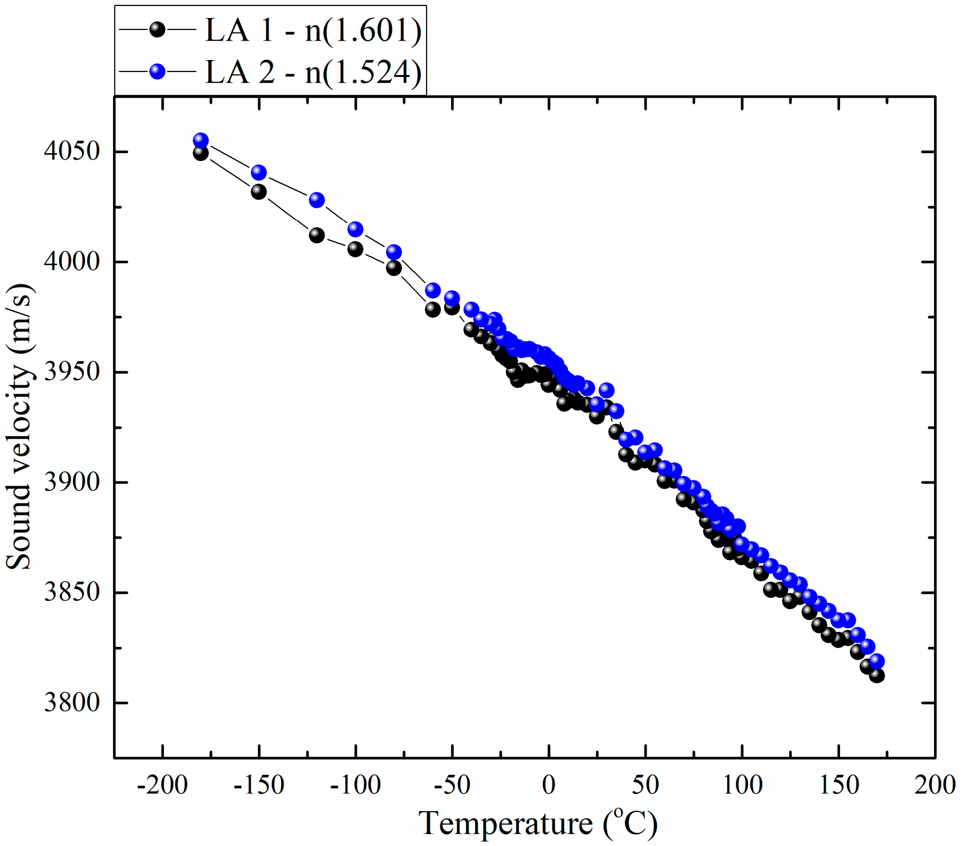


**Supplementary Figure S1.** Temperature dependence of the sound velocities of the LA1 and LA2 modes obtained by using the theoretically predicted refractive indices [22] and Eq.(4) as described in the text.
